# Supplementary material for: Spinal muscular atrophy-like phenotype in a mouse model of acid ceramidase deficiency
Source: Commun Biol. 2023 May 25;6:560. doi: 10.1038/s42003-023-04932-w (PMC10212955; doi:10.1038/s42003-023-04932-w)
Supplement: Supplementary file 2 — Supplementary Information [file 42003_2023_4932_MOESM2_ESM.pdf]

Spinal Muscular Atrophy-Like Phenotype in a Mouse Model of Acid Ceramidase Deficiency

Supplementary Information

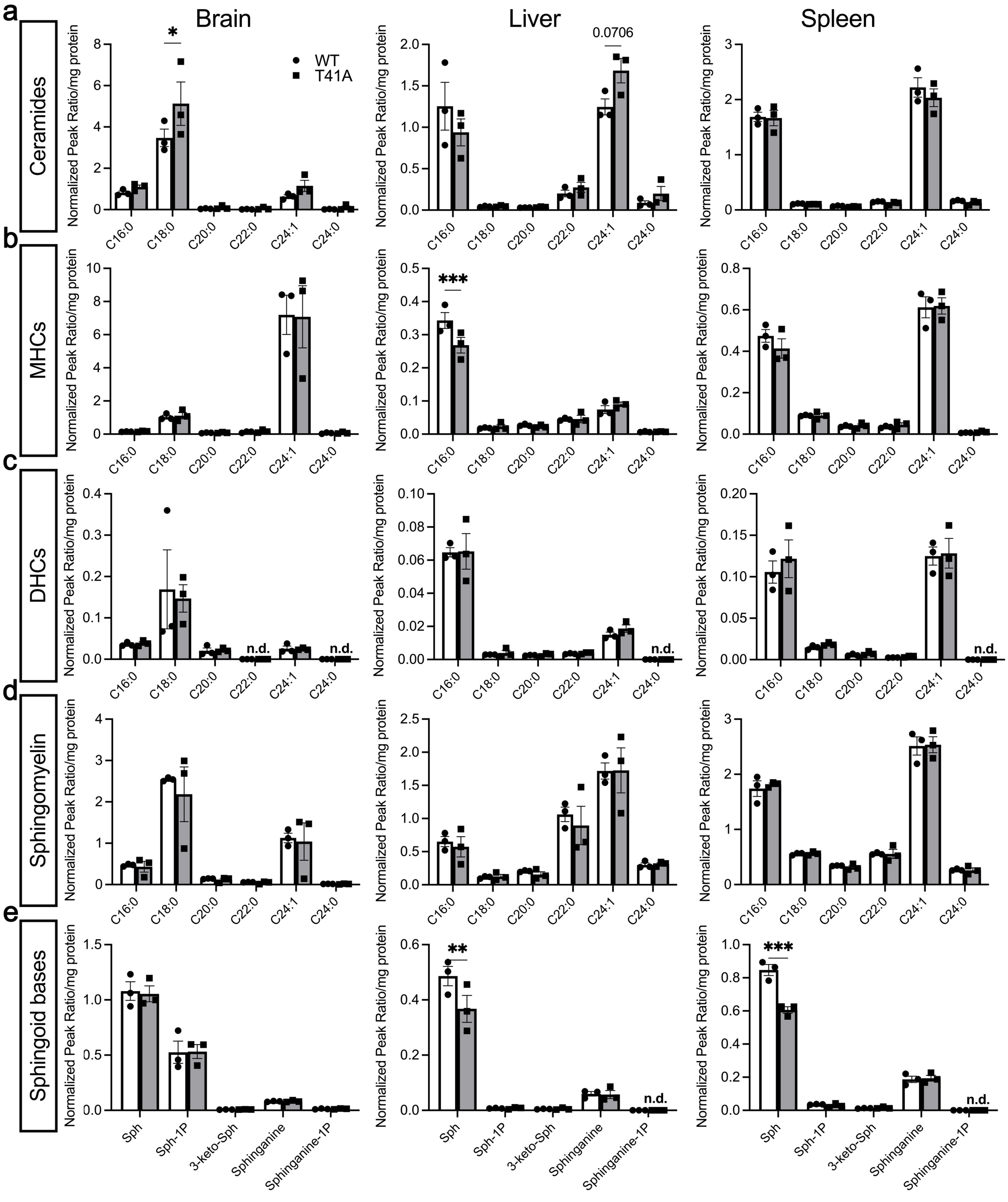

Supplementary Figure 1: T41A mutant mice do not accumulate pathological levels of sphingolipids. (**a-e**) Lipids were extracted from brains, livers and spleens of T41A homozygous mutant and wild-type (WT) mice aged ~21 months. Levels of 6 acyl-chain variants, as indicated, of ceramide (a), monohexosylceramides (MHCs, b), dihexosylceramides (DHCs, c), sphingomyelin (d) and sphingoid bases (e) namely sphingosine (Sph), Sph-1-phosphate (Sph-1P), 3-keto-Sph, Sphinganine, and

Sphinganine-1P were quantified by mass spectrometry. Points reflect values obtained for individual mice and bars depict means (n=3) of peak area ratios (analyte versus standard) normalized to protein concentration. Error bars are standard error of the mean. Data were compared using a two-way analysis of variance with Bonferroni's multiple comparisons test, *** $p<0.001$, ** $p<0.01$ and * $p<0.05$. Abbreviations – n.d.: not detected

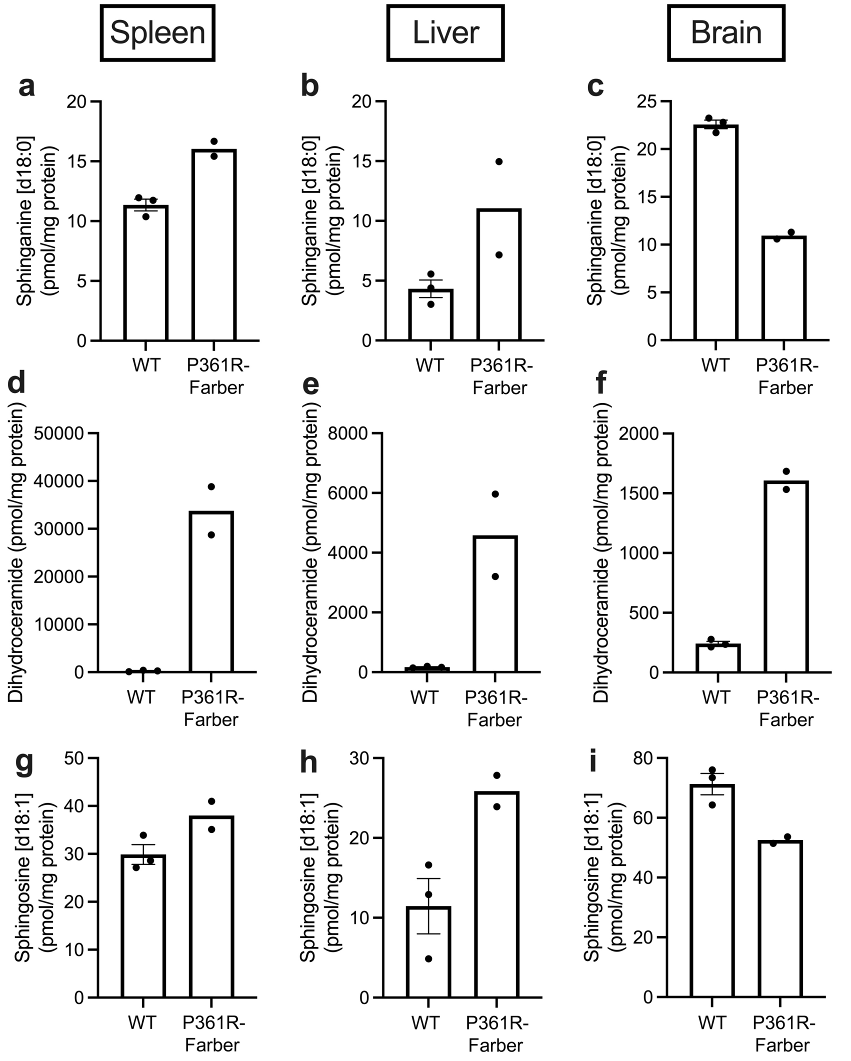

Supplementary Figure 2: Changes in sphinganine, dihydroceramide, and sphingosine levels in homogenates from P361R-Farber spleens, livers, and brains. (**a-c**) Sphinganine levels in spleen (a), liver (b), and whole brain (c) homogenates from wild-type (WT; n=3) and P361R-Farber mice (n=2). Average sphingolipid levels are shown as bars, points show values for individual mice (WT, n=3; P361R-Farber, n=2), and error bars show the standard error of the mean. (**d-f**) Dihydroceramide levels in spleen (d), liver (e), and brain (f) homogenates from WT and P361R-Farber mice. Total amount of dihydroceramide was calculated by summing molar amounts of individually measured species. Data are presented as in (a-c). (**g-i**) Sphingosine levels in spleen (g), liver (h), and brain (i) homogenates from WT and P361R-Farber mice. Data are presented as in (a-c).

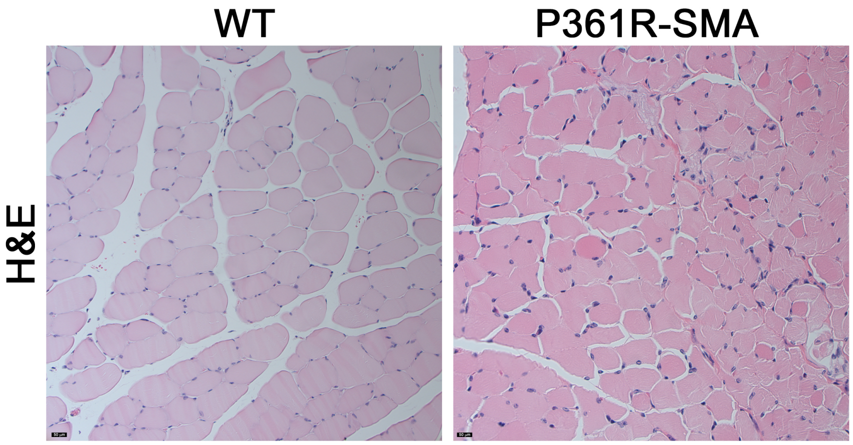

Supplementary Figure 3: Histological examination of muscle. Sections from the posterior thigh muscles of WT and P361R-SMA mice were stained using H&E. Scale bars represent 50µm.

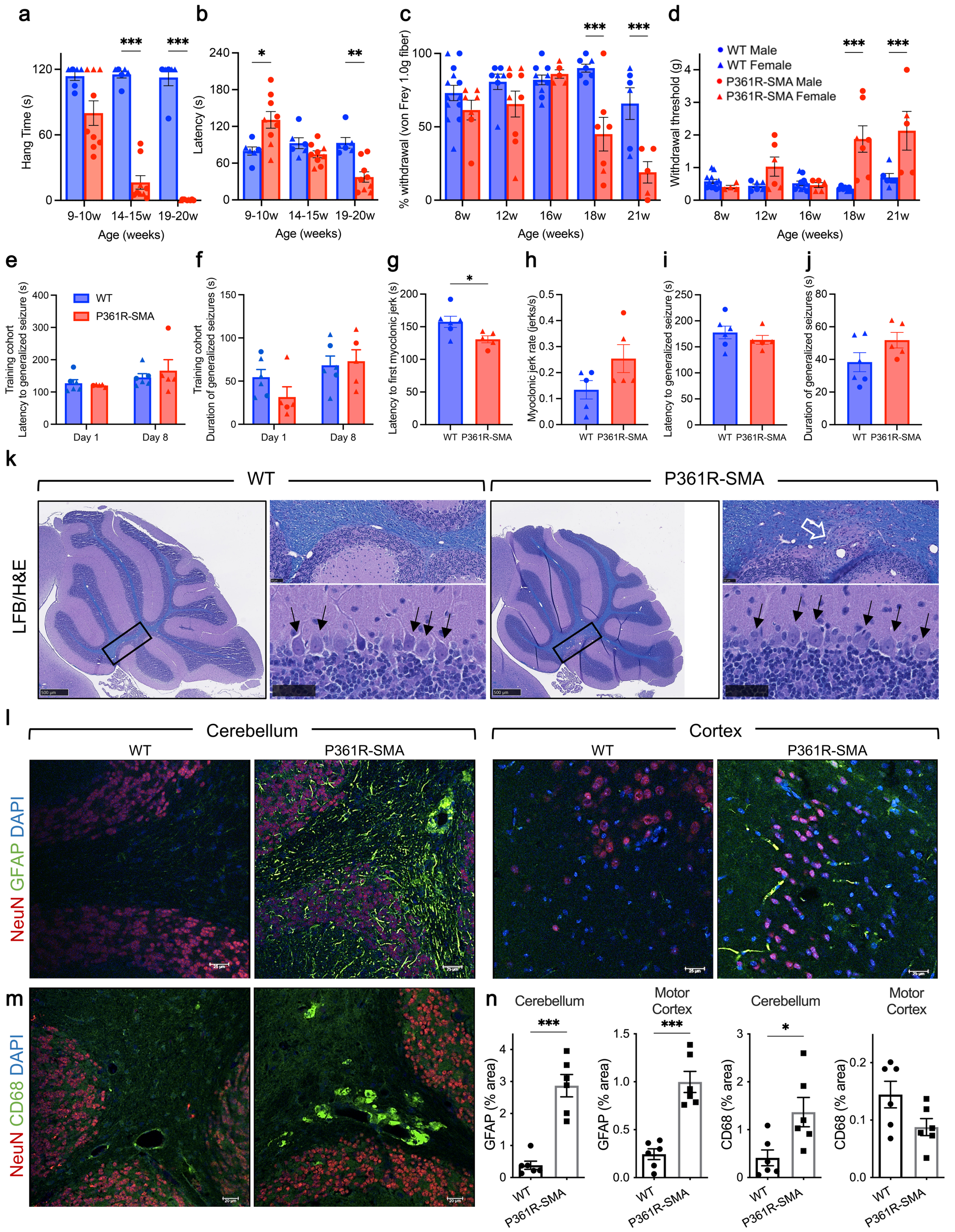

a
Hang Time (s)
9-10w 14-15w 19-20w
Age (weeks)
b
Latency (s)
9-10w 14-15w 19-20w
Age (weeks)
c
% withdrawal (von Frey 1.0g fiber)
8w 12w 16w 18w 21w
Age (weeks)
d
Withdrawal threshold (g)
WT Male
WT Female
P361R-SMA Male
P361R-SMA Female
8w 12w 16w 18w 21w
Age (weeks)
e
Training cohort Latency to generalized seizure (s)
WT
P361R-SMA
Day 1 Day 8
f
Training cohort Duration of generalized seizures (s)
Day 1 Day 8
g
Latency to first myoclonic jerk (s)
WT P361R-SMA
h
Myoclonic jerk rate (jerks/s)
WT P361R-SMA
i
Latency to generalized seizure (s)
WT P361R-SMA
j
Duration of generalized seizures (s)
WT P361R-SMA
k
WT
P361R-SMA
LFB/H&E
l
Cerebellum
Cortex
WT
P361R-SMA
NeuN GFAP DAPI
m
NeuN CD68 DAPI
n
Cerebellum
Motor Cortex
GFAP (% area)
CD68 (% area)
WT
P361R-SMA

Supplementary Figure 4: Evaluation of sensory-motor behavior in P361R-SMA mice, and gross structure, myelin, and inflammation in 21-23-week-old P361R-SMA cerebella and motor cortices. (**a-b**) Wire-hang (a) and rotarod (b) test performance of wild-type (WT) and P361R-SMA mice. Bars represent mean values (n=6 WT and n=9 P361R-SMA), error bars depict standard error of the mean, and points show measurements for individual mice where triangles are females and circles are males. (**c**) Percent withdrawal response of WT and P361R-SMA mice to mechanical hind paw stimulus by a 1.0g von Frey filament. Bars represent mean values (n=13, 7, 10, 7 and 6 for WT, and n=6, 9, 5, 7, 5 for P361R-SMA mice for the 5 time points indicated), error bars depict standard error of the mean, and points show measurements for individual mice where triangles are females and circles are males. (**d**) Paw-withdrawal force threshold for WT and P361R-SMA mice determined using incremental increase in von Frey filament rigidity. Bars represent mean values (n=12, 6, 10, 7 and 6 for WT, and n=4, 6, 5, 7 and 5 for P361R-SMA for the 5 time points indicated), error bars depict standard error of the mean, and points show measurements for individual mice where triangles are females and circles are males. Data in (a-d) were compared using two-way analyses of variance with Šidák's multiple comparisons, *** $p<0.001$, ** $p<0.01$ and * $p<0.05$. (**e-j**) Flurothyl-induced seizure behaviors in P361R-SMA and wild-type (WT) mice. The latency to generalized seizure (e) and duration of generalized seizure (f) were recorded on training days 1 and 8 of exposure to flurothyl. Mice were rechallenged (retest) after a 28-day incubation phase. The latency to the first myoclonic jerk (threshold; g), myoclonic jerk frequency (h), latency to generalised seizures (i) and duration of generalized seizures (j) were recorded during the rechallenge phase. Bars represent mean values (n=6 WT and n=5 P361R-SMA mice), error bars depict standard error of the mean, and points show measurements for individual mice where triangles are females and circles are males. Data were compared using Welch's t-tests, * $p<0.05$. (**k**) Sections from cerebella of WT and P361R-SMA mice stained with luxol-fast blue (LFB) and H&E; the scale bars represent 500µm. A higher magnification of the boxed region is shown to the right (top panel), along with a close-up of Purkinje cells (bottom panel); the scale bars represent 50µm. (**l,m**) Representative images of immunofluorescence staining for activated astrocytes (l) or microglia/macrophages (m) and neurons (NeuN) in the cerebellum and/or motor cortex. The scale bars represent 25µm. (**n**) Quantification of immunofluorescence staining in (l,m). Immunofluorescence was quantified as % area in 3 random images from 2 mice of each genotype and values plotted as individual points. The mean value is indicated with error bars showing standard error of the mean. Values were compared using Welch's t-tests, *** $p<0.001$, and * $p<0.05$. Abbreviations – GFAP: Glial fibrillary acidic protein.

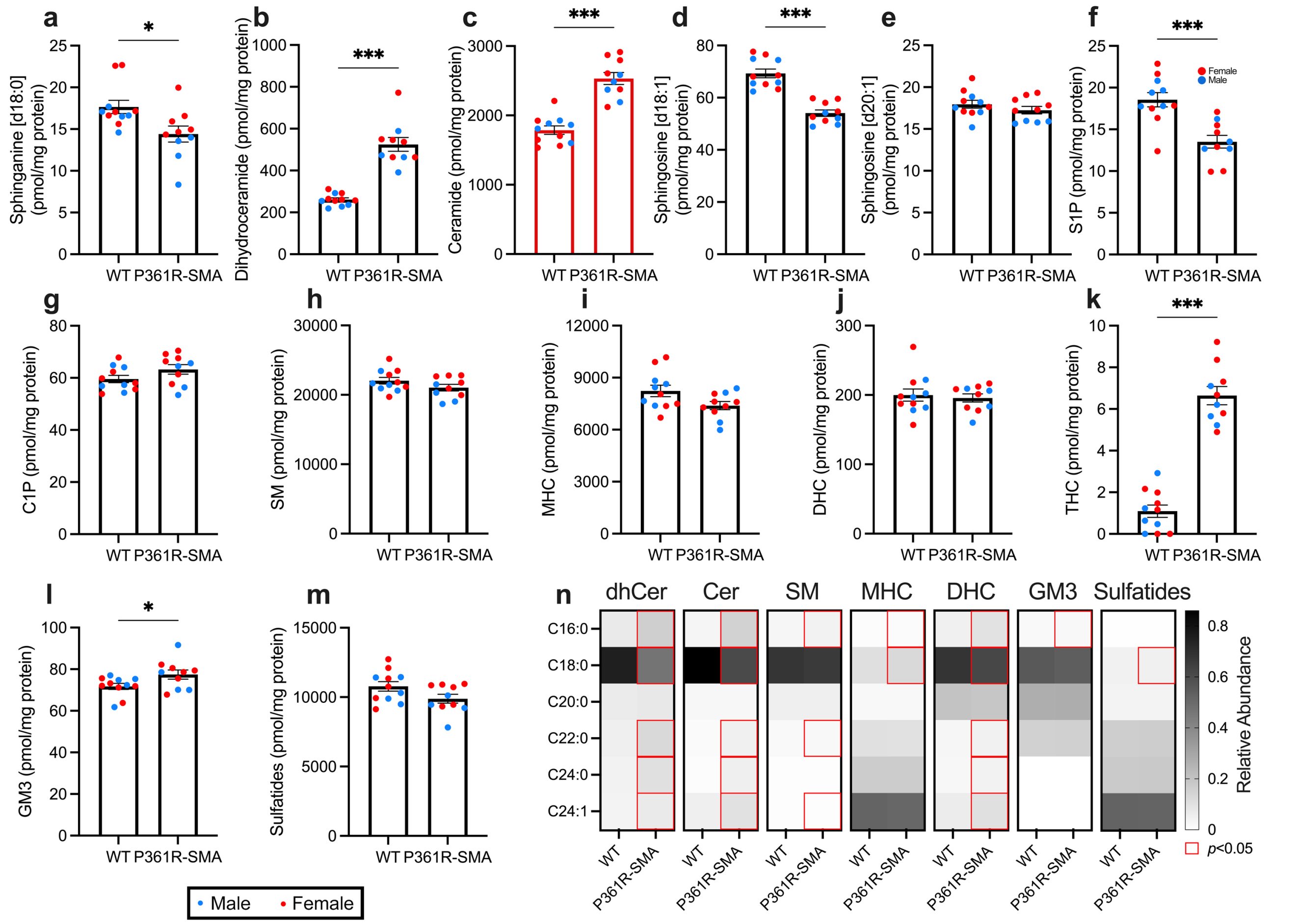

Supplementary Figure 5: Ceramides (Cer), dihydroceramides (dhCer) and other sphingolipid levels in whole brain lysates from 21-week-old P361R-SMA mice. (**a-m**) Brains were homogenized, and mass spectrometry was used to measure levels of the indicated sphingolipids. Total amounts of each sphingolipid family were calculated by adding up molar amounts of individually measured acyl-chain variants. Values from individual mice (WT, n=11; P361R-SMA, n=10) are shown as points, sex is denoted using blue for males and red for females, bars indicate the mean values, and error bars show standard error of the mean. Ceramide levels are highlighted with red bars. Data were compared using Welch's t-tests, *** $p<0.001$, ** $p<0.01$ and * $p<0.05$. (**n**) Proportions of acyl-chain variants within the indicated families of sphingolipids are shown as heatmaps for comparison. Statistically significant ($p<0.05$) differences in relative abundances, indicated with a red outline, were determined using a two-way analysis of variance with Šidák's multiple comparison. Abbreviations – WT: wild-type; S1P: sphingosine-1-phosphate; C1P: ceramide-1-phosphate; SM: sphingomyelin; MHC: monohexosylceramide; DHC: dihexosylceramide; THC: trihexosylceramide; GM3: monosialodihexosylganglioside

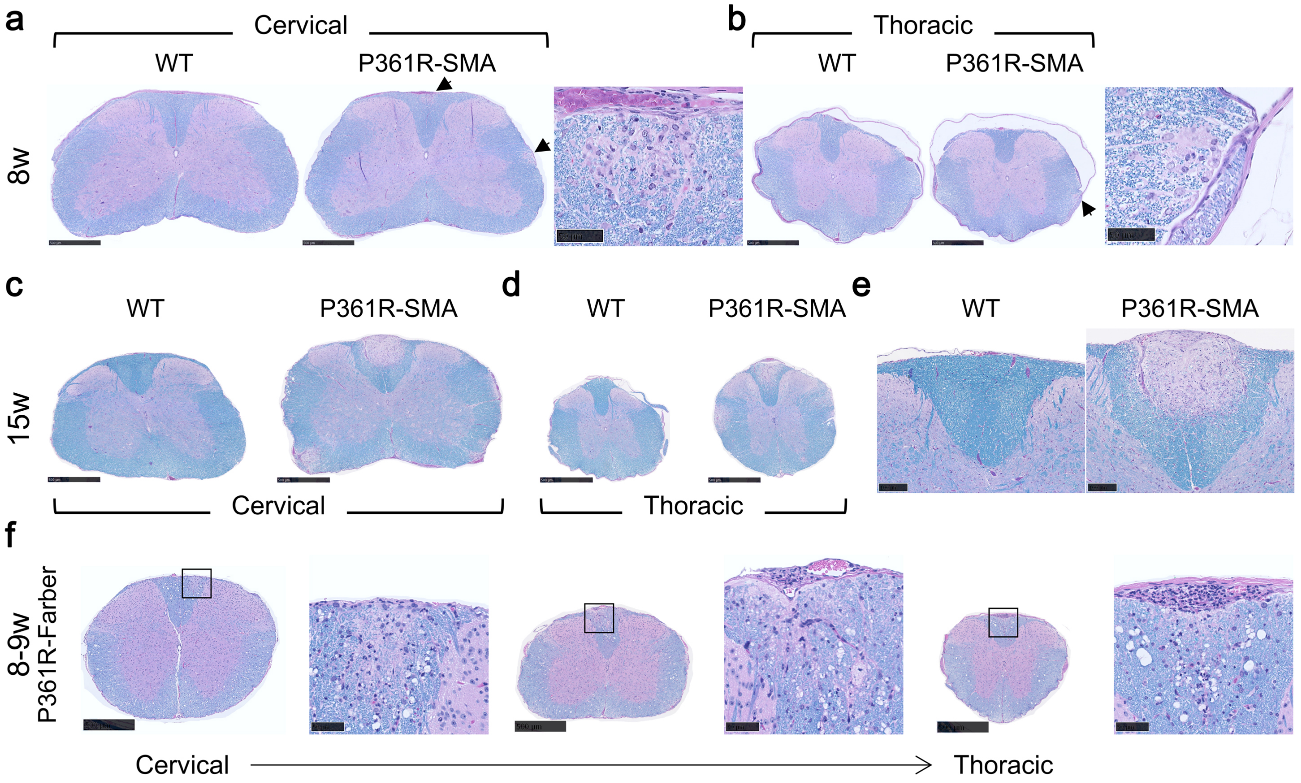

Supplementary Figure 6: Spinal cord pathology in 8w and 15w-old P361R-SMA mice, and in moribund P361R-Farber mice. (**a,b**) Sections from cervical (a) and thoracic (b) spinal cord of 8-week-old wild-type (WT) or P361R-SMA mice stained with luxol-fast blue and hematoxylin & eosin (LFB/H&E). The scale bars represent 500µm. A higher magnification of the suspected lesion is shown to the right in each case; the scale bar represents 50µm. (**c-e**) Sections from cervical (c) and thoracic (d) spinal cord of 15-week-old WT or P361R-SMA mice stained with LFB/H&E. The scale bars represent 500µm. A higher magnification of a lesion in the dorsal column is shown in (e); the scale bar represents 100µm. (**f**) Representative sections from moribund P361R-Farber mice stained with LFB/H&E. The scale bars represent 500µm. A higher magnification of suspected lesions (boxed) is shown to the right; the scale bar represents 50µm.

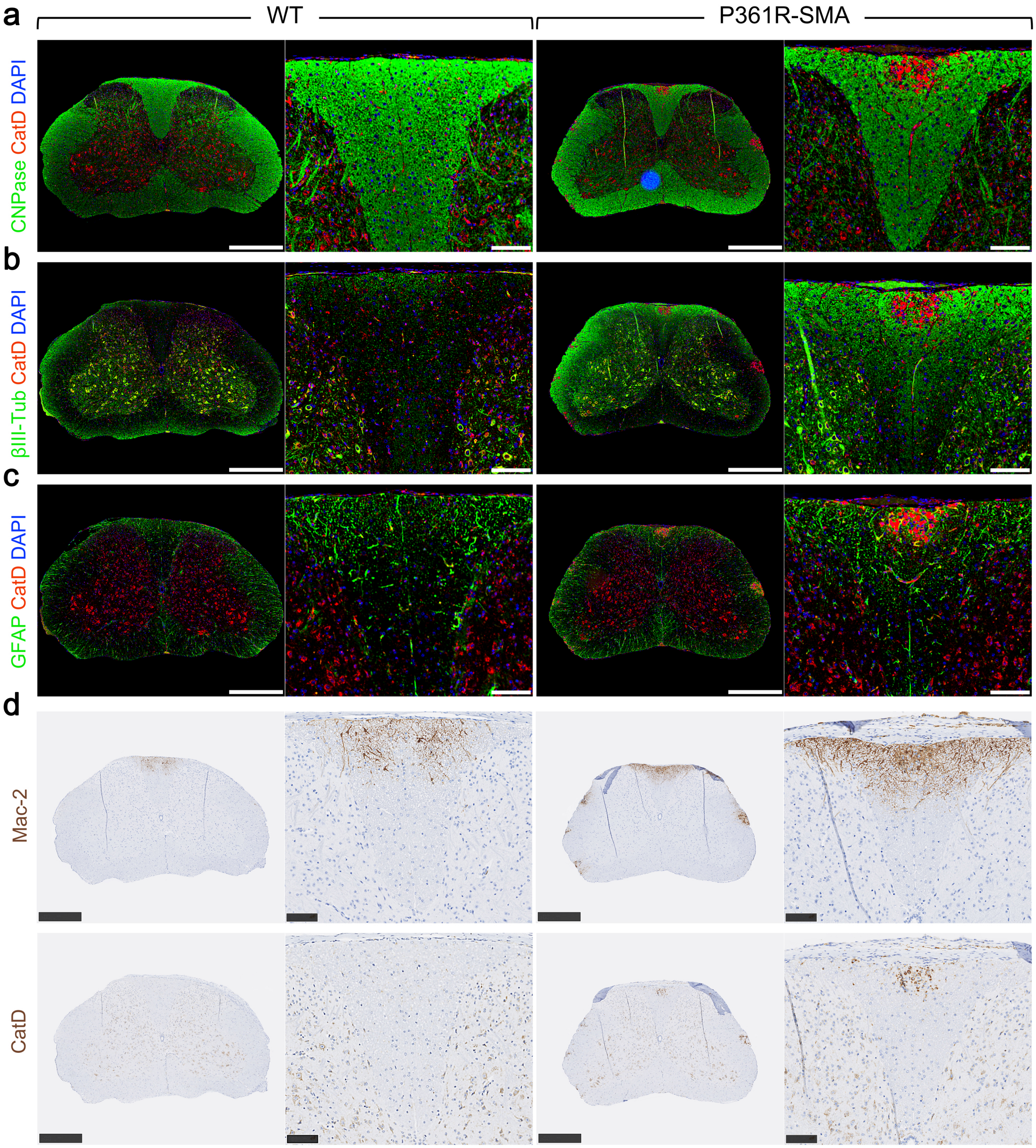

Supplementary Figure 7: Immunofluorescent staining for myelin, neurons and phagocytic cells in spinal cord sections from 8-9-week-old P361R-SMA mice. (**a,b**) Staining for CNPase, a myelin-associated protein (a), neuron-specific βIII-Tubulin (βIII-Tub) (b) along with CathepsinD (CatD) in spinal cord sections from wild-type (WT) and P361R-SMA mice. The scale bars represent 500μm (left) and 100μm (right). (**c**) Astrocytes (GFAP) and lysosomes (CatD) were immunostained in spinal cord sections from WT and P361R-SMA mice. The scale bars represent 500μm (left) and 100μm (right). (**d**) Serial sections from spinal cords of WT and P361R-SMA mice were immunohistochemically stained for phagocytic macrophages/microglia (Mac-2) and CatD. The scale bars represent 500μm (left) and 100μm (right).

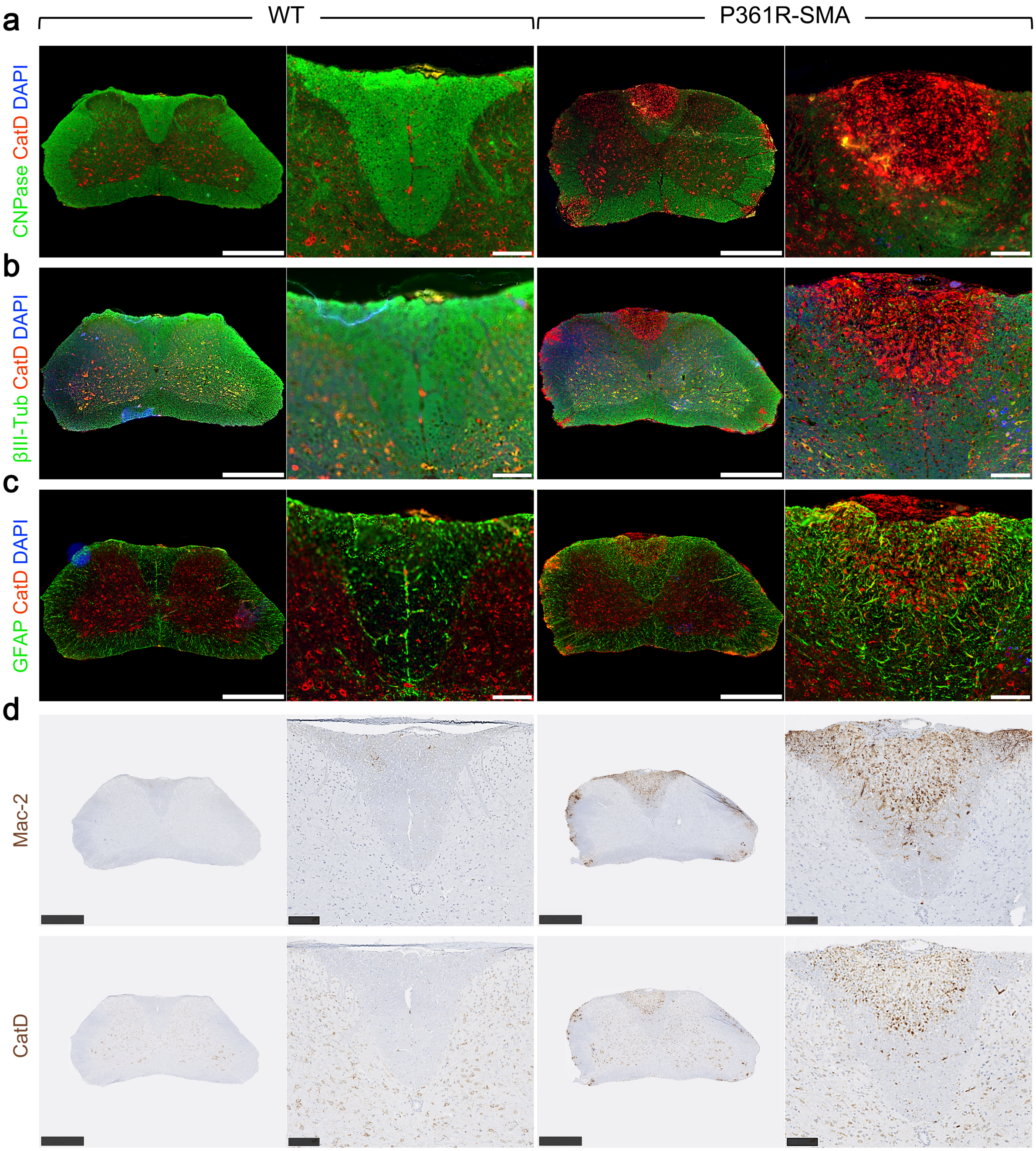

Supplementary Figure 8: Immunofluorescent staining for myelin, neurons and phagocytic cells in spinal cord sections from 15-week-old P361R-SMA mice. (**a,b**) Staining for CNPase, a myelin-associated protein (a), neuron-specific βIII-Tubulin (βIII-Tub) (b) along with CathepsinD (CatD) in spinal cord sections from wild-type (WT) and P361R-SMA mice. The scale bars represent 500µm (left) and 100µm (right). (**c**) Astrocytes (GFAP) and lysosomes (CatD) were immunostained in spinal cord sections from WT and P361R-SMA mice. The scale bars represent 500µm (left) and 100µm (right). (**d**) Serial sections from spinal cords of WT and P361R-SMA mice were immunohistochemically stained for phagocytic macrophages/microglia (Mac-2) and CatD. The scale bars represent 500µm (left) and 100µm (right).

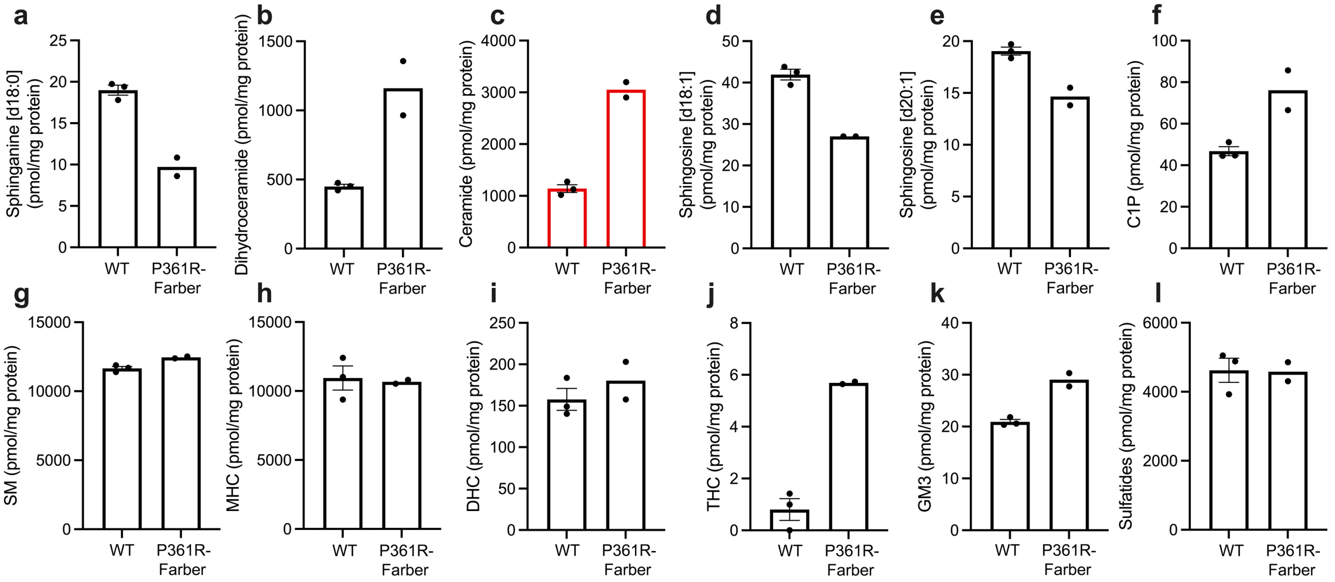

Supplementary Figure 9: Ceramide (Cer) and related sphingolipid levels in spinal cord homogenates from 7-8-week-old P361R-Farber mice. (**a-l**) Spinal cords from P361R-Farber mice were resected from the cervical to mid-lumbar regions, and the indicated sphingolipids quantified in homogenates. Total sphingolipids levels were calculated by summing the molar amounts of individual acyl-chain variants. Plotted points in a-l reflect levels in individual mice (WT, n=3; P361R-Farber, n=2), averages are shown as bars, and error bars show the standard error of the mean. Ceramide levels are highlighted with red bars. Abbreviations – dhCer: dihydroceramide; C1P: ceramide-1-phosphate; SM: sphingomyelin; MHC: monohexosylceramide; DHC: dihexosylceramide; THC: trihexosylceramide; GM3: monosialodihexosylganglioside

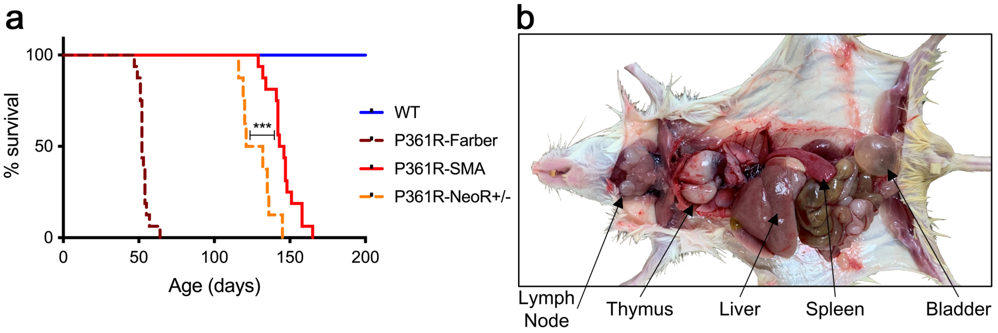

Supplementary Figure 10: Lifespan and post-mortem examination of P361R-Farber and P361R-SMA compound mutants (P361R-NeoR+/-). (**a**) Kaplan-Meier survival curve for P361R-NeoR+/- mice shown relative to P361R-Farber and P361R-SMA mice. Survival curves for P361R-SMA (n=16) and P361R-NeoR+/- (n=8) mice were compared using the Kaplan-Meier estimator (Log-rank test), *** $p<0.001$. (**b**) A representative photograph of a post-mortem dissection of a P361R-NeoR+/- mouse. Visceral organs with pathology reminiscent of P361R-Farber mice are labelled. Photograph of the dissected mouse was taken post-mortem using an Apple iPhone XS Max with default settings.

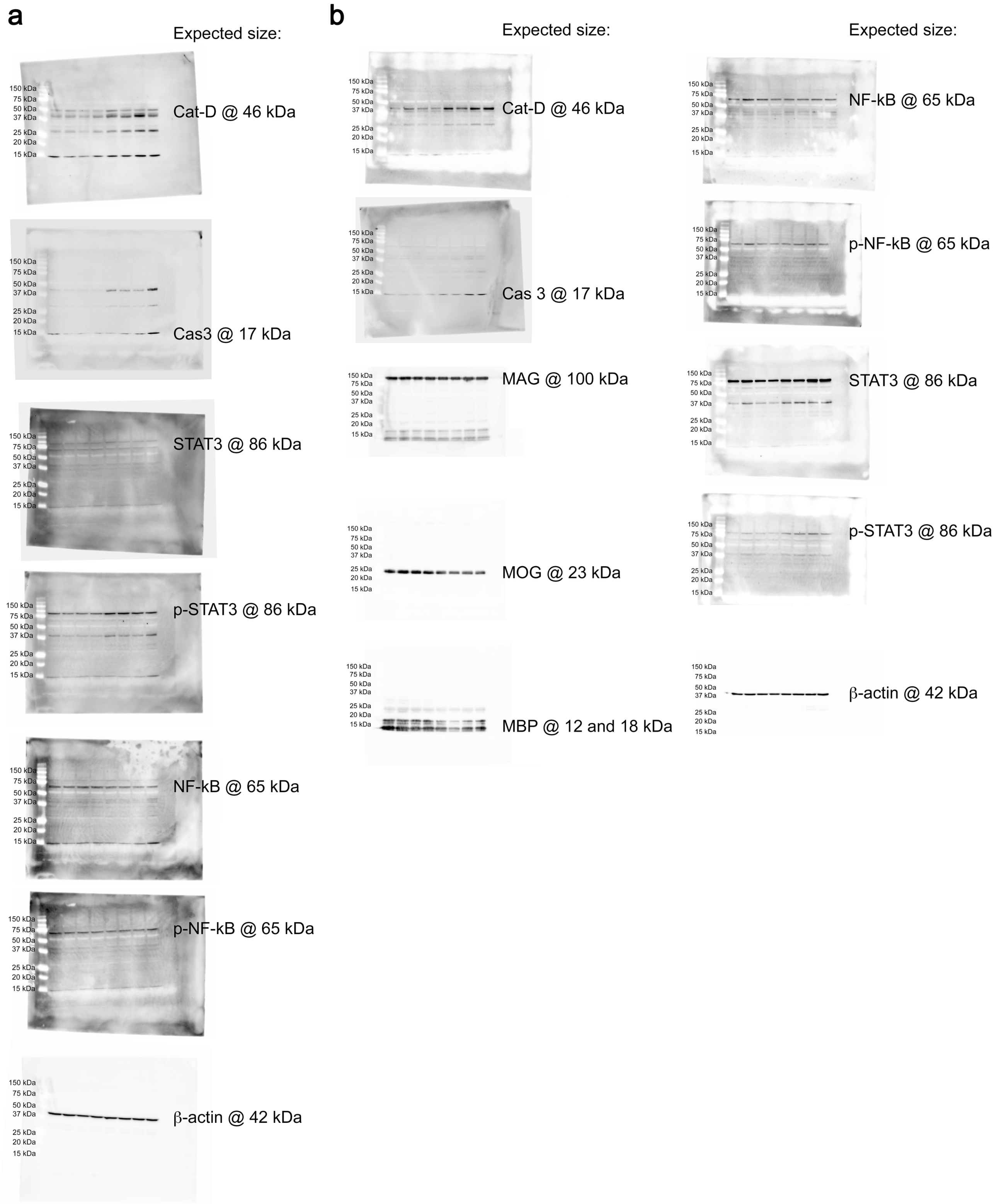

Supplementary Figure 11: Uncropped and unedited western blots with annotated molecular weight and expected band size used in Figure 2 and Figure 5. a) Full western blot membranes for Cat-D, Cas3, STAT3, p-STAT3, NF-kB, p-NF-kB and β-actin shown in Figure 2. b) Full western blot membranes for Cat-D, Cas3, MAG, MOG, MBP, NF-kB, p-NF-kB, STAT3, p-STAT3 and β-actin shown in Figure 5.

Supplementary Table 1: Multiple reaction monitoring transitions and analyte dependant parameters for phosphorylated sphingolipids.

Lipid	MRM	RT (min)	DP	EP	CE	CXP
S1P (d17:1) IS	366.2/250.2	1.9	50	10	20	15
S1P (d18:1)	380.3/264.4	2.3	40	10	21	12
C1P (d18:1/12:0) IS	562.4/264.4	4.2	60	10	36	20
C1P (d18:1/16:0)	618.5/264.4	5.1	60	10	36	20
C1P (d18:1/18:0)	646.5/264.4	5.6	45	10	42	20
C1P (d18:1/20:0)	674.5/264.4	5.8	45	10	42	20
C1P (d18:1/22:0)	702.6/264.4	6.0	45	10	42	20
C1P (d18:1/24:0)	730.6/264.4	6.3	45	10	42	20
C1P (d18:1/24:1)	728.6/264.4	6.0	45	10	42	20

C1P, ceramide-1-phosphate; CE, collision energy; CXP, collision exit potential; DP, declustering potential; EP, entrance potential; IS, internal standard; MRM, multiple reaction monitoring; RT, retention time; S1P, sphingosine-1-phosphate.
