# Supplementary material for: Spinal muscular atrophy-like phenotype in a mouse model of acid ceramidase deficiency
Source: Commun Biol. 2023 May 25;6:560. doi: 10.1038/s42003-023-04932-w (PMC10212955; doi:10.1038/s42003-023-04932-w)
Supplement: Supplementary file 3 — Description of Additional Supplementary Files [file 42003_2023_4932_MOESM3_ESM.pdf]

Description of Additional Supplementary Files

File name: Supplementary Data 1

Description: Source data for all statistical analyses organized by Figure.

File name: Supplementary Data 2

Description: Comparison of individual acyl-chain variants of the indicated sphingolipid species between tissue extracts from wild-type (WT) and P361R-SMA mice. Mean levels for each genotype are indicated (WT, n=11; P361R-SMA, n=10) and were compared using pairwise Welch's t-tests. Fold-changes are relative to WT means and are shaded green and red corresponding to being increased or decreased in tissues from P361R-SMA mice, respectively. False discovery rate-adjusted p-values are indicated. Only ACVs detected in at least one animal are shown. Individual sphingomyelin isoforms cannot be resolved, so they are indicated as total number of carbons and unsaturated bonds in the sphingoid backbone and acyl-chain.

File name: Supplementary Data 3

Description: Statistical analysis by sex.

File name: Supplementary Data 4

Description: Results of statistical analyses organized by Figure.

File name: Supplementary Movie 1

Description: Representative videos of 21-26-week-old P361R-SMA mice with advanced SMA-like phenotypes. Videos show mice are active but have tremors, hind limb paralysis, and urine scalding in the genital area due to incontinence.
