# Supplementary material for: Spinal muscular atrophy-like phenotype in a mouse model of acid ceramidase deficiency
Source: Commun Biol. 2023 May 25;6:560. doi: 10.1038/s42003-023-04932-w (PMC10212955; doi:10.1038/s42003-023-04932-w)
Supplement: Supplementary file 9 — Reporting Summary [file 42003_2023_4932_MOESM9_ESM.pdf]

nature portfolio

Corresponding author(s): Jeffrey A Medin

Last updated by author(s): May 6, 2023

Reporting Summary

Nature Portfolio wishes to improve the reproducibility of the work that we publish. This form provides structure for consistency and transparency in reporting. For further information on Nature Portfolio policies, see our Editorial Policies and the Editorial Policy Checklist.

Statistics

For all statistical analyses, confirm that the following items are present in the figure legend, table legend, main text, or Methods section.

n/a	Confirmed	
☐	☒	The exact sample size (*n*) for each experimental group/condition, given as a discrete number and unit of measurement
☐	☒	A statement on whether measurements were taken from distinct samples or whether the same sample was measured repeatedly
☐	☒	The statistical test(s) used AND whether they are one- or two-sided<br>*Only common tests should be described solely by name; describe more complex techniques in the Methods section.*
☐	☒	A description of all covariates tested
☐	☒	A description of any assumptions or corrections, such as tests of normality and adjustment for multiple comparisons
☐	☒	A full description of the statistical parameters including central tendency (e.g. means) or other basic estimates (e.g. regression coefficient) AND variation (e.g. standard deviation) or associated estimates of uncertainty (e.g. confidence intervals)
☐	☒	For null hypothesis testing, the test statistic (e.g. *F*, *t*, *r*) with confidence intervals, effect sizes, degrees of freedom and *P* value noted<br>*Give P values as exact values whenever suitable.*
☒	☐	For Bayesian analysis, information on the choice of priors and Markov chain Monte Carlo settings
☒	☐	For hierarchical and complex designs, identification of the appropriate level for tests and full reporting of outcomes
☒	☐	Estimates of effect sizes (e.g. Cohen's *d*, Pearson's *r*), indicating how they were calculated

Our web collection on statistics for biologists contains articles on many of the points above.

Software and code

Policy information about availability of computer code

Data collection	QuantStudio software (Applied Biosystems v1.3)<br>SkanIt Software (Thermo Scientific v2.6)
Data analysis	QuantStudio software (Applied Biosystems v1.3)<br>NDP.view2 software (Hamamatsu Photonics v2.9)<br>ImageJ (NIH v1.51)<br>MultiQuant (SCIEX v3.0.1)<br>OlyVIA software (Olympus v3.2.1)<br>paint.net (dotPDN, LLC v4.2.15)<br>MRIcroGL software (University of South Carolina, v1.2.20191219)<br>GraphPad Prism (GraphPad Software, LLC v9.1.0)

For manuscripts utilizing custom algorithms or software that are central to the research but not yet described in published literature, software must be made available to editors and reviewers. We strongly encourage code deposition in a community repository (e.g. GitHub). See the Nature Portfolio guidelines for submitting code & software for further information.

March 2021

Data

Policy information about availability of data

All manuscripts must include a data availability statement. This statement should provide the following information, where applicable:

- Accession codes, unique identifiers, or web links for publicly available datasets
- A description of any restrictions on data availability
- For clinical datasets or third party data, please ensure that the statement adheres to our policy

This study includes no data deposited in external repositories

Field-specific reporting

Please select the one below that is the best fit for your research. If you are not sure, read the appropriate sections before making your selection.

☒ Life sciences ☐ Behavioural & social sciences ☐ Ecological, evolutionary & environmental sciences

For a reference copy of the document with all sections, see nature.com/documents/nr-reporting-summary-flat.pdf

Life sciences study design

All studies must disclose on these points even when the disclosure is negative.

Sample size	An n of 3-5 was chosen for quantitative studies for each sex, except western blotting (n=2 per sex). Values were compared between sexes to see if they were different. When substantially different, they were reported independently (e.g. weight in Figure 1). Otherwise, data from different sexes were pooled. When pooled, n were large enough (6-11) to confidently report statistical comparisons. For qualitative studies, consistency between 3 animals was evaluated per group. No other tests were done to determine sample size.
Data exclusions	Data were not excluded from analyses
Replication	All studies were done with samples from individual mice, obtained from different litters, and reproducibility between sexes was also evaluated. Western blots were conducted twice using independent samples to confirm findings. Mass spectrometry was conducted with technical replicates for each tissue homogenate from each mouse. Major histological findings were independently confirmed by an external service provider (Charles Rivers).
Randomization	Mice were obtained randomly from various litters as available.
Blinding	Mice were assigned IDs and measurements for the most part were taken by blinded investigators; note that it is difficult for studies involving live mice or intact tissues to have proper blinding, as mutant mice/samples are easy to spot.

Reporting for specific materials, systems and methods

We require information from authors about some types of materials, experimental systems and methods used in many studies. Here, indicate whether each material, system or method listed is relevant to your study. If you are not sure if a list item applies to your research, read the appropriate section before selecting a response.

Materials & experimental systems

n/a	Involved in the study	
☐	☒	Antibodies
☐	☒	Eukaryotic cell lines
☒	☐	Palaeontology and archaeology
☐	☒	Animals and other organisms
☒	☐	Human research participants
☒	☐	Clinical data
☒	☐	Dual use research of concern

Methods

n/a	Involved in the study	
☒	☐	ChIP-seq
☒	☐	Flow cytometry
☐	☒	MRI-based neuroimaging

Antibodies

Antibodies used	anti-Cathepsin D (ab75852, Abcam), anti-Caspase-3 (9664S), anti-STAT3 (4904), anti-p-STAT3 (9145), anti-NF-κB (8242), anti-p-NF-κB (3033), anti-MBP (78896S), anti-MOG (96457S) and anti-MAG (9043T, all from Cell Signaling Technologies), anti-β-Actin (a3854, Sigma-Aldrich), anti-Rabbit IgG (A6154, Sigma–Aldrich), anti-Mac-2 clone M3/38 (Cedarlane CL8942AP), anti-Lamp1 clone D2D11 (Cell Signaling 9091S), anti-Cathepsin D clone EPR3057Y (Abcam ab75852), Cathepsin D (clone EPR3057Y, Abcam ab75852), CNPase (clone CL2887, Novus Biological NBP2-46617), βIII-Tubulin (clone TuJ-1, R&D Systems MAB1195SP), GFAP (clone GA5, Cell Signaling 3670T), anti-mouse neuronal nuclear antigen (NeuN) (1:200) (E4M5P; Cell Signaling Technology), rabbit polyclonal anti-mouse/rat

	CD68 (1:200) (AB125212; Abcam), rabbit anti-mouse/rat NeuN (1:200) (MABN140; Millipore), and mouse anti-mouse/rat GFAP (1:100) (8-1E7; DSHB)
Validation	Unless otherwise indicated, all antibodies were used as recommended by the manufacturer and results compared to those previously found in the literature for validity, or tested on different tissue for which we know the expected result.

Eukaryotic cell lines

Policy information about cell lines

Cell line source(s)	HEK293T - ATCC; Ear skin fibroblasts - made
Authentication	Ear skin fibroblasts were genotyped. HEK293T cells were not authenticated.
Mycoplasma contamination	Ear skin fibroblasts were not tested for mycoplasma, HEK293T tested negative
Commonly misidentified lines (See ICLAC register)	N/A

Animals and other organisms

Policy information about studies involving animals; ARRIVE guidelines recommended for reporting animal research

Laboratory animals	Mouse->C57BL/6J->Male&Female->3-26weeks of age for P361R-SMA and up to 2yrs of age for T41A
Wild animals	N/A
Field-collected samples	N/A
Ethics oversight	MCW IACUC

Note that full information on the approval of the study protocol must also be provided in the manuscript.

Magnetic resonance imaging

Experimental design

Design type	Conducted on fixed tissue
Design specifications	Conducted on fixed tissue
Behavioral performance measures	N/A

Acquisition

Imaging type(s)	Structural
Field strength	9.4T
Sequence & imaging parameters	A fast spin echo sequence was used for three-dimensional imaging at an isotropic resolution of 55µm3 (192x96x96 matrix). Two echo times (23.5 and 86ms) were acquired with a 4000ms repetition time, turbo factor of 8, and 4 repetitions for a total imaging time of about 5.5hrs per sample.
Area of acquisition	Fixed spinal column sections (6-7mm) were imaged ex vivo

Diffusion MRI ☐ Used ☒ Not used

Preprocessing

Preprocessing software	Data were converted to NIfTI-1 data format using MRIcroGL software (v1.2.20191219, University of South Carolina)
Normalization	Images from separate repetitions were spatially aligned to account for any scanner drift or movement of the sample during the scan using advanced normalization tools (ANTs)
Normalization template	Samples were normalized within repeats
Noise and artifact removal	Images from separate repetitions were spatially aligned to account for any scanner drift or movement of the sample during the scan using advanced normalization tools (ANTs). The eight images from the two echoes and four repetitions were averaged to increase signal-to-noise.
Volume censoring	N/A

Statistical modeling & inference

Model type and settings	N/A
Effect(s) tested	N/A

Specify type of analysis: ☐ Whole brain ☐ ROI-based ☐ Both

Statistic type for inference (See Eklund et al. 2016)	N/A
Correction	N/A

Models & analysis

n/a	Involved in the study
☒	☐ Functional and/or effective connectivity
☒	☐ Graph analysis
☒	☐ Multivariate modeling or predictive analysis
